# Glutamate alleviates intestinal injury, maintains mTOR and suppresses TLR4 and NOD signaling pathways in weanling pigs challenged with lipopolysaccharide

**DOI:** 10.1038/s41598-018-33345-7

**Published:** 2018-10-11

**Authors:** Qin Qin, Xiao Xu, Xiuying Wang, Huanting Wu, Huiling Zhu, Yongqing Hou, Bing Dai, Xiuting Liu, Yulan Liu

**Affiliations:** 1 Hubei Key Laboratory of Animal Nutrition and Feed Science, Hubei Collaborative Innovation Center for Animal Nutrition and Feed Safety, Wuhan Polytechnic University, Wuhan, 430023 P. R. China; 2 Zhe Jiang Goshine Test Technologies Co., Ltd., Hangzhou, 310030 P. R. China

## Abstract

This experiment aimed to explore whether glutamate (Glu) had beneficial effects on intestinal injury caused by *Escherichia coli* LPS challenge via regulating mTOR, TLRs, as well as NODs signaling pathways. Twenty-four piglets were allotted to 4 treatments including: (1) control group; (2) LPS group; (3) LPS + 1.0% Glu group; (4) LPS + 2.0% Glu group. Supplementation with Glu increased jejunal villus height/crypt depth ratio, ileal activities of lactase, maltase and sucrase, and RNA/DNA ratio and protein abundance of claudin-1 in jejunum and ileum. In addition, the piglets fed Glu diets had higher phosphorylated mTOR (Ser^2448^)/total mTOR ratio in jejunum and ileum. Moreover, Glu decreased TNF-α concentration in plasma. Supplementation with Glu also decreased mRNA abundance of jejunal TLR4, MyD88, IRAK1, TRAF6, NOD2 and increased mRNA abundance of ileal Tollip. These results indicate that Glu supplementation may be closely related to maintaining mTOR and inhibiting TLR4 and NOD signaling pathways, and concomitant improvement of intestinal integrity under an inflammatory condition.

## Introduction

The intestine is not only critical for the digestion and absorption of nutrients, but also interacts with a complex external milieu^1^. As the largest component of innate immune system, the intestine employs a number of unique strategies to defend against bacterial-derived endogenous and exogenous harmful agents^2^. However, the intestinal health status could be hazarded by a lot of factors including infection and inflammation^3,4^. Inflammation can potentially leads to intestinal injury and dysfunction which can result in decreased animal performance and health^5^. Therefore, maintaining intestinal health is vital for humans and animals.

In weaned piglets, conditions that compromise the integrity of the small intestine may be related to processes of the production of proinflammatory cytokines such as tumor necrosis factor-*α* (TNF-*α*)^6^, which could be induced by activation of inflammatory signaling pathways such as toll-like receptors (TLRs) and nucleotide-binding oligomerization domain proteins (NODs)^7,8^. TLRs and NODs are important protein families that participate in recognizing pathogen-associated molecular patterns (PAMPs) and modulating congenital antibacterial and inflammatory responses^7,9^. In the TLRs family, TLR4 is a critical receptor in transduction of the inflammatory response and plays an important role in intestinal homeostasis^10^. Meanwhile, NOD1 and NOD2 are prominent members in NODs family, whose roles in innate immunity and inflammatory diseases have been studied^11^, particularly in intestine^12,13^. After interacting with their specific PAMPs, activated TLRs or NODs trigger downstream signaling pathways that lead to activation of nuclear factor-κB (NF-κB), which further motivates the expression of pro-inflammatory cytokines, including interleukin (IL)-1*β*, IL-6 and TNF-*α*^7,8^. Therefore, exploring nutrients to improve intestinal integrity through inhibiting intestinal TLR4 and NOD signaling pathways further reducing inflammatory response has been a research hotspot in the human or animal nutrition areas.

Mammalian target of rapamycin (mTOR) is a serine-threonine kinase which is related to several important aspects of mammalian cell function^14^. The activity of mTOR is regulated by various extracellular and intracellular factors such as amino acids, energy and hormones, in turn, mTOR changes rates of translation, transcription, protein synthesis and degradation, cell signaling, metabolism as well as cytoskeleton dynamics^15–17^. Many researchers have presented that activation of mTOR signaling pathway could improve intestinal health status including enhanced intestinal barrier function in Caco-2 cells^18^, and improved cell proliferation and protein synthesis in intestinal porcine epithelial cells-J2 (IPEC-J2)^15,19^. Therefore, exploring a nutritional additive to improve intestinal integrity by activating mTOR signaling is also a critical issue which needs to be solved.

Glutamate (Glu), as an acidic amino acid, is traditionally classified as a non-essential amino acid. As the research developed, some studies have shown that Glu has some important functions in nutrition, metabolism and organ health status. Firstly, Glu is one of the major metabolic fuels for small intestine to maintain function and protect mucosal integrity^20^. In addition, it is important in stabilizing the protein structure^21^ and activating taste receptors in the digestive tract^21,22^. Moreover, Glu is a member of the “arginine family”, which comprises arginine, glutamine, glutamate, proline, aspartate, asparagine, ornithine, and citrulline. These amino acids are interconvertible via complex interorgan metabolism to play important roles in the body^23^. Some studies showed that dietary supplementation with Glu improved intestinal health status^23,24^. Rezaei *et al*. reported that monosodium glutamate supplementation maintained the small-intestinal structure and function in weaned pigs^24^. Jiao *et al*. reported that Glu enhanced barrier and antioxidative functions in intestinal porcine epithelial cells^25^. These studies are mostly focused on the beneficial effect of Glu on intestinal morphology, barrier and antioxidative capacity, however, few reports about the mechanisms underlying the protective effect of Glu are found.

Accordingly, we hypothesized that Glu may ameliorate intestinal injury induced by lipopolysaccharide (LPS) through regulating mTOR, TLR4 as well as NOD signaling pathways. The aim of our experiment was to explore whether Glu could attenuate LPS-induced intestinal injury, and to reveal its molecular mechanism(s).

## Results

### Intestinal morphology

**Figure 1:**
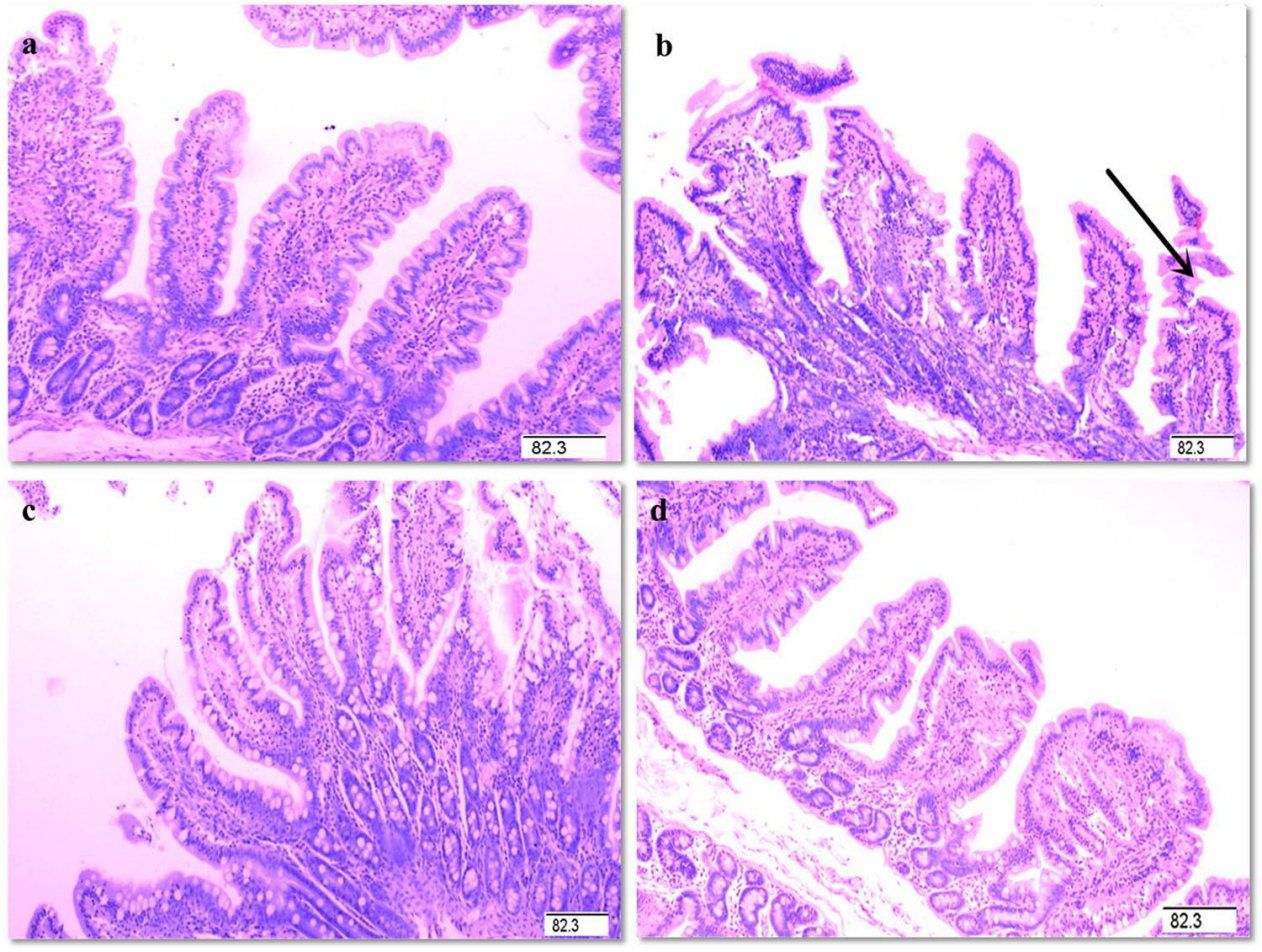
Histological appearance of jejunal mucosa (hematoxylin and eosin). (**a**) control. (**b**) LPS. Arrow represents the damaged intestinal mucosa in the LPS-challenged pigs. (**c**) LPS + 1.0% Glu. (**d**) LPS + 2.0% Glu. Original magnification 100×. Scale bars = 82.6 μm.

The intestinal mucosal condition in control piglets was good (Fig. 1). However, the LPS-challenged pigs exhibited intestinal injury. After supplementation with Glu, the intestinal mucosal injury was alleviated. Compared with the pigs in LPS group, supplementation with 2.0% Glu increased villus height/crypt depth ratio (VCR, Table 1) in jejunum (p < 0.05).

**Table 1:** Effects of dietary supplementation with Glu on intestinal morphology of weanling piglets after 4 h *Escherichia coli* LPS challenge.

Item	Control	LPS	LPS + 1.0% Glu	LPS + 2.0% Glu	SEM	p value
Jejunum						
Villus height (μm)	289	285	285	274	14	0.731
Crypt depth (μm)	106	102	95	94	6	0.148
Villus height/crypt depth ratio	2.79^c^	2.82^bc^	2.89^ab^	2.94^a^	0.05	0.014
Ileum						
Villus height (μm)	241	240	265	232	17	0.285
Crypt depth (μm)	83	81	87	78	6	0.487
Villus height/crypt depth ratio	2.92	2.91	3.03	2.97	0.05	0.081

Data are the means of 6 replicates with 1 piglet per replicate. Means with the same row without common superscripts differ (p < 0.05).

### Intestinal protein, DNA and RNA contents

LPS challenge reduced the ratio of RNA to DNA in ileum compared with the pigs in control group (p < 0.05, Table 2). Among the LPS-challenged pigs, supplementation of 2.0% Glu increased the ratio of RNA to DNA in jejunum (p < 0.05). Supplementation with 1.0% Glu increased the ratio of RNA to DNA in ileum (p < 0.05).

**Table 2:** Effects of dietary supplementation with Glu on intestinal mucosal protein, DNA and RNA concentrations of weanling piglets after 4 h *Escherichia coli* LPS challenge.

Item	Control	LPS	LPS + 1.0% Glu	LPS + 2.0% Glu	SEM	p value
Jejunum						
Protein (mg/g tissue)	84.8	82.8	89.4	86.6	5.7	0.691
RNA/DNA (μg/μg)	3.2^b^	3.3^b^	4.1^ab^	5.2^a^	0.6	0.026
Protein/DNA (mg/μg)	0.198	0.168	0.220	0.205	0.021	0.125
Ileum						
Protein (mg/g tissue)	58.2	61.8	60.6	64.5	3.2	0.287
RNA/DNA (μg/μg)	10.2^a^	7.6^b^	9.9^a^	8.4^ab^	0.9	0.018
Protein/DNA (mg/μg)	0.310	0.240	0.303	0.250	0.033	0.101

Data are the means of 6 replicates with 1 piglet per replicate. Means with the same row without common superscripts differ (p < 0.05).

### Intestinal disaccharidase activities

LPS challenge decreased the activity of maltase in jejunum, as well as the activities of maltase and sucrase in ileum of the piglets (p < 0.05, Table 3). However, supplementation with 1.0% Glu increased the activities of maltase and sucrase in ileum compared with pigs in LPS group (p < 0.05). Moreover, supplementation with 2.0% Glu increased the activities of lactase compared with other groups (p < 0.05).

**Table 3:** Effects of dietary supplementation with Glu on disaccharidase activities of weanling piglets after 4 h *Escherichia coli* LPS challenge.

Item	Control	LPS	LPS + 1.0% Glu	LPS + 2.0% Glu	SEM	p value
Jejunum						
Lactase (U/mg protein)	15.6	14.6	7.9	12.9	5.1	0.459
Maltase (U/mg protein)	169^a^	75^b^	92^b^	97^b^	19	<0.001
Sucrase (U/mg protein)	47.3	38.7	25.2	29.6	10	0.160
Ileum						
Lactase (U/mg protein)	1.6^b^	1.3^b^	2.7^b^	4.4^a^	0.7	0.001
Maltase (U/mg protein)	85^a^	56^b^	74^a^	51^b^	8	0.001
Sucrase (U/mg protein)	11.6^a^	7.7^b^	10.3^a^	7.2^b^	1.1	0.002

Data are the means of 6 replicates with 1 piglet per replicate. Means with the same row without common superscripts differ (p < 0.05).

### Intestinal claudin-1 protein expression

**Figure 2:**
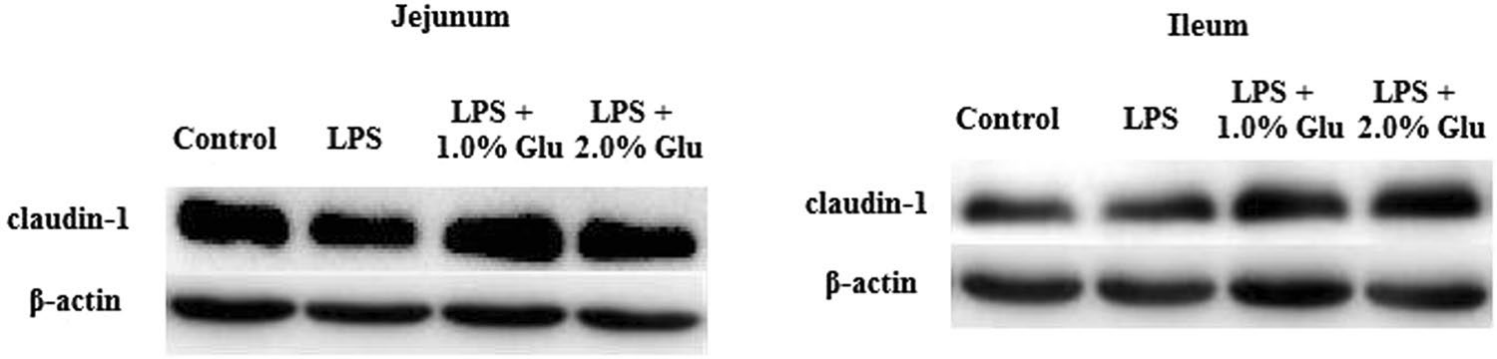
Western blotting analysis of the expression of clauding-1 in the jejunal and ileal mucosa. n = 6.

No difference was observed in intestinal claudin-1 protein expression between control and LPS-challenged piglets (Table 4). Supplementation with Glu increased the protein expression of claudin-1 both in jejunum and ileum compared with the pigs in LPS group (Fig. 2, p < 0.05).

**Table 4:** Effects of dietary supplementation with Glu on tight junction protein expression in the intestinal mucosa of weanling piglets after 4 h *Escherichia coli* LPS challenge.

Item	Control	LPS	LPS + 1.0% Glu	LPS + 2.0% Glu	SEM	p value
Jejunum						
Claudin-1/β-actin	0.202^b^	0.246^b^	0.331^a^	0.328^a^	0.033	0.002
Ileum						
Claudin-1/β-actin	0.531^c^	0.653^bc^	0.730^b^	0.974^a^	0.087	0.002

Data are the means of 6 replicates with 1 piglet per replicate. Means with the same row without common superscripts differ (p < 0.05).

### Plasma TNF-α concentration

Compared with control piglets, LPS challenge increased TNF-α concentration in plasma (p < 0.05, Table 5). Supplementation with 2.0% Glu decreased plasma TNF-α concentration in piglets compared with LPS group piglets (p < 0.05).

**Table 5:** Effects of dietary supplementation with Glu on TNF-α concentration in plasma after 4 h *Escherichia coli* LPS challenge in weaned pigs.

Item	Control	LPS	LPS + 1.0% Glu	LPS + 2.0% Glu	SEM	p value
Plasma TNF-α, pg/ml	49^c^	2793^a^	1578^ab^	1029^bc^	679	0.006

Data are the means of 6 replicates with 1 piglet per replicate. Means with the same row without common superscripts differ (p < 0.05).

### Intestinal protein abundances of key signaling molecules in mTOR signaling pathway

**Figure 3:**
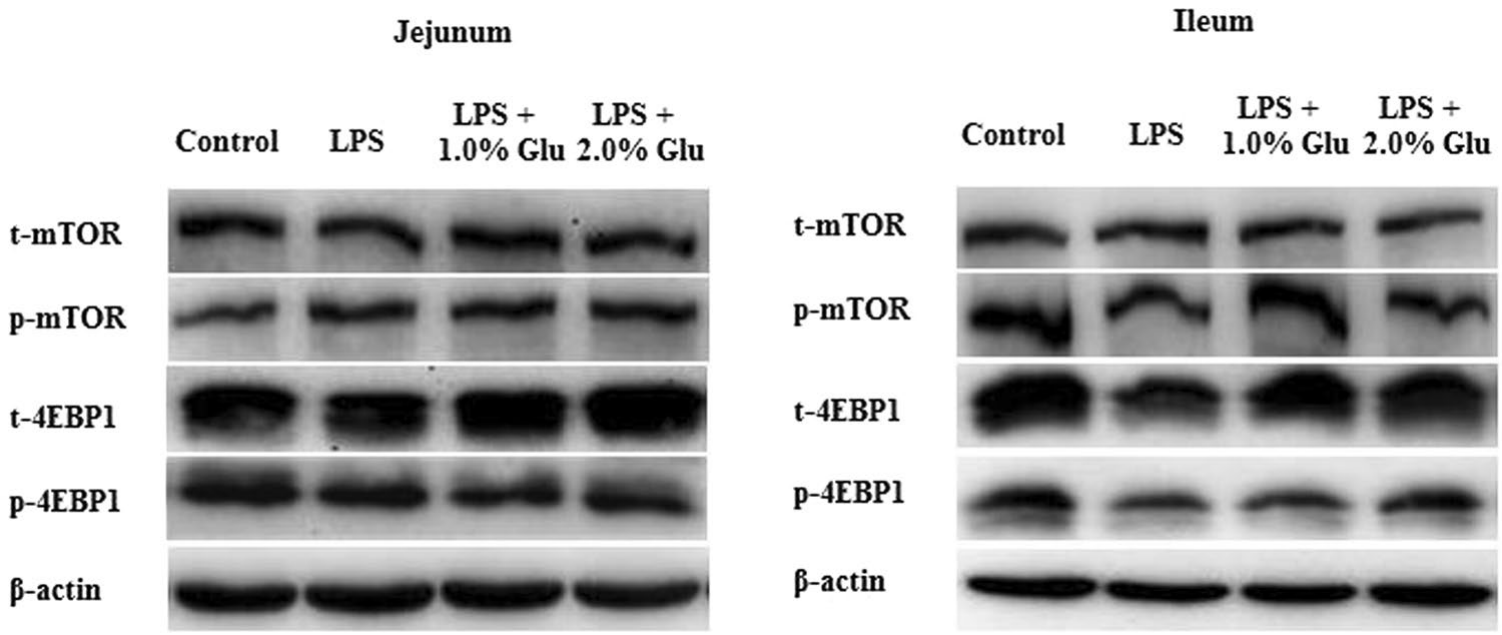
Western blotting analysis of the expression of mTOR and 4EBP1 in the jejunal and ileal mucosa. n = 6.

The piglets challenged with LPS had decreased phosphorylated mTOR (Ser^2448^) (p-mTOR, p < 0.05) and phosphorylated 4E-binding protein 1 (Thr^70^) (p-4EBP1, p < 0.05) protein expression in ileum (Table 6, Fig. 3). The ratio of p-4EBP1/t-4EBP1 protein expression in ileum of LPS-challenged pigs was also reduced compared with pigs in control group (p < 0.05). Compared with the pigs in LPS group, supplementation with 2.0% Glu increased jejunal ratio of p-mTOR/t-mTOR (p < 0.05) and supplementation with 1.0% Glu increased ileal ratio of p-mTOR/t-mTOR (p < 0.05).

**Table 6:** Effects of dietary supplementation with Glu on protein expression of proteins related to mTOR signaling pathway in the intestinal mucosa of weanling piglets after 4 h *Escherichia coli* LPS challenge.

Item	Control	LPS	LPS + 1.0% Glu	LPS + 2.0% Glu	SEM	p value
Jejunum						
t-mTOR	42.2	37.6	45.2	50.0	7.9	0.473
p-mTOR	21.7	22.3	25.5	27.9	4.9	0.557
p-mTOR/t-mTOR	0.54^b^	0.58^b^	0.48^b^	0.73^a^	0.07	0.006
t-4EBP1	21.0	27.9	26.3	23.5	5.6	0.634
p-4EBP1	10.0	8.0	5.8	5.8	2.6	0.338
p-4EBP1/t-4EBP1	0.34	0.27	0.18	0.28	0.08	0.276
Ileum						
t-mTOR	12.7	11.1	10.2	11.1	2.1	0.673
p-mTOR	15.6^a^	11.8^b^	14.5^ab^	12.3^b^	1.5	0.062
p-mTOR/t-mTOR	1.29^b^	1.11^b^	1.79^a^	1.27^b^	0.23	0.042
t-4EBP1	30.1	21.2	30.3	31.8	4.8	0.145
p-4EBP1	36.0^a^	16.4^b^	19.3^b^	28.1^ab^	6.2	0.017
p-4EBP1/t-4EBP1	1.22^a^	0.77^b^	0.62^b^	0.90^ab^	0.17	0.020

Data are the means of 6 replicates with 1 piglet per replicate. Means with the same row without common superscripts differ (p < 0.05).

### Intestinal mRNA expression of key signaling molecules in TLR4 and NODs signaling pathways

The LPS-challenged pigs had increased mRNA expression of TLR4, Myeloid differentiation protein 2 (MD2), cluster of differentiation 14 (CD14), myeloid differentiation factor 88 (MyD88), IL-1 receptor-associated kinase 1 (IRAK1), TNF receptor associated factor 6 (TRAF6), NOD2, receptor-interacting serine/threonine-protein kinase 2 (RIPK2), NF-κB in jejunum, and NOD2 and RIPK2 in ileum compared with control piglets (p < 0.05, Table 7). Compared with pigs in LPS group, supplementation with 2.0% Glu reduced mRNA expression of jejunal TLR4, MyD88, IRAK1, TRAF6, and NOD2 (p < 0.05).

**Table 7:** Effects of dietary supplementation with Glu on mRNA expression of key genes related to TLR4 and NODs signaling pathways in the intestinal mucosa of weanling piglets after 4 h *Escherichia coli* LPS challenge.

Item	Control	LPS	LPS + 1.0% Glu	LPS + 2.0% Glu	SEM	p value
Jejunum						
TLR4	1.00^c^	1.85^a^	1.64^ab^	1.33^bc^	0.21	0.004
MD2	1.00^b^	1.97^a^	1.91^a^	1.83^a^	0.21	0.001
CD14	1.00^b^	2.16^a^	2.75^a^	2.32^a^	0.41	0.003
LBP	1.00	0.88	0.84	0.60	0.22	0.336
MyD88	1.00^b^	1.25^a^	1.30^a^	0.98^b^	0.09	0.004
IRAK1	1.00^bc^	1.16^a^	1.03^ab^	0.87^c^	0.06	0.002
TRAF6	1.00^bc^	1.32^a^	1.17^ab^	0.93^c^	0.09	0.003
NOD1	1.00^a^	0.70^ab^	0.87^a^	0.46^b^	0.14	0.007
NOD2	1.00^b^	2.32^a^	1.97^a^	1.33^b^	0.21	<0.001
RIPK2	1.00^b^	1.99^a^	1.83^a^	1.64^a^	0.17	<0.001
NF-κB	1.00^b^	1.41^a^	1.29^a^	1.18^ab^	0.11	0.012
Ileum						
TLR4	1.00	1.28	1.25	1.12	0.13	0.152
MD2	1.00^b^	1.33^a^	1.21^ab^	1.32^a^	0.14	0.087
CD14	1.00^b^	1.13^b^	1.44^ab^	1.69^a^	0.22	0.022
LBP	1.00	1.18	1.13	0.62	0.35	0.388
MyD88	1.00^b^	1.10^ab^	1.20^a^	0.97^b^	0.06	0.010
IRAK1	1.00	0.87	0.90	0.91	0.06	0.261
TRAF6	1.00^b^	1.12^ab^	1.32^a^	1.12^ab^	0.11	0.062
NOD1	1.00	1.07	0.89	0.83	0.12	0.241
NOD2	1.00^b^	2.06^a^	2.19^a^	1.69^ab^	0.35	0.013
RIPK2	1.00^b^	2.01^a^	2.02^a^	1.85^a^	0.19	< 0.001
NF-κB	1.00	1.13	1.11	1.04	0.09	0.513

Data are the means of 6 replicates with 1 piglet per replicate. Means with the same row without common superscripts differ (p < 0.05).

### Intestinal mRNA expression of TLR4 and NODs negative regulators

Compared with the pigs in control group, LPS challenge decreased mRNA expression of toll-interacting protein (Tollip), and Erbb2 interacting protein (ERBB2IP) in ileum (p < 0.05, Table 8). Supplementation with 2.0% Glu enhanced mRNA expression of Tollip in ileum (p < 0.05). However, the mRNA expressions of ERBB2IP in jejunum were decreased with the supplementation with 2.0% Glu (p < 0.05).

**Table 8:** Effects of dietary supplementation with Glu on mRNA expression of negative regulating factors related to TLR4 and NODs signaling pathways in the intestinal mucosa of weanling piglets after 4 h E*scherichia coli* LPS challenge.

Item	Control	LPS	LPS + 1.0% Glu	LPS + 2.0% Glu	SEM	p value
Jejunum						
RP105	1.00	2.09	2.19	1.44	0.62	0.210
SOCS1	1.00^b^	2.98^a^	2.56^a^	2.21^a^	0.46	0.002
Tollip	1.00	0.61	0.74	0.93	0.18	0.145
SIGIRR	1.00	1.00	0.93	0.97	0.06	0.634
ERBB2IP	1.00^a^	0.96^a^	0.91^a^	0.74^b^	0.08	0.014
ACAP1	1.00^a^	0.84^ab^	0.88^a^	0.66^b^	0.10	0.018
Ileum						
RP105	1.00	0.76	0.89	1.01	0.23	0.663
SOCS1	1.00^b^	1.11^b^	1.56^a^	1.07^b^	0.19	0.028
Tollip	1.00^a^	0.64^b^	0.90^ab^	1.13^a^	0.15	0.025
SIGIRR	1.00	1.07	1.07	0.96	0.10	0.632
ERBB2IP	1.00^a^	0.80^b^	0.93^ab^	0.88^ab^	0.07	0.049
ACAP1	1.00	0.78	0.78	0.80	0.10	0.113

Data are the means of 6 replicates with 1 piglet per replicate. Means with the same row without common superscripts differ (p < 0.05).

## Discussion

Glu is one of the most abundant amino acids in alimentary proteins^26^. The majority of Glu molecule is either oxidized as energy or metabolized into other nonessential amino acids via transamination^27^. Recently, some studies showed that Glu plays an important role in maintaining physiological function as well as energy level in intestine^28^. In the present study, Glu improved the intestinal integrity and function via maintaining mTOR, as well as suppressing TLR4 and NOD signaling pathways in the piglets under an inflammation condition.

The intestinal healthy status is often reflected by both structural and functional integrity^4^. Villus height, crypt depth and VCR have been used to quantify the intestinal morphology^29^. Mucosal protein content is an important indicator for cell metabolism, and protein deficiency negatively affects the intestinal epithelial barrier of piglets^30^. RNA/DNA ratio reflects the cell capacity for protein synthesis^31^. Protein/DNA ratio in mucosa has been employed as an indicator of intestinal growth and repair^4^. Mucosal disaccharidases, namely lactase, maltase and sucrase, reflect intestinal digestive function^32^. Claudins are major constituents of tight junctions^33^. In present study, the piglets challenged with LPS had decreased RNA/DNA ratio, and maltase and sucrase activities, which is similar to the findings of Liu *et al*. and Wang *et al*.^4,34^. These results indicate that injection of LPS caused intestinal injury in weaned pigs. Glu supplementation to the LPS-challenged pigs increased jejunal VCR. Besides, supplementation with Glu increased RNA/DNA ratio as well as protein expression of claudin-1 in jejunum and ileum. Furthermore, the pigs fed Glu diets had enhanced ileal disaccharidase activities. These results indicate that Glu had a significant effect on protecting intestinal mucosa and improving intestinal repair. Similarly, there were several studies that showed results in accordance with our findings. Rezaei *et al*. reported that monosodium glutamate supplementation increased villus height and DNA content in weaned pigs^24^. In addition, Lin *et al*. found that Glu supplementation increased the relative mRNA expression of occludin and zonula occludens protein-1 in jejunal mucosa in weaning piglets^35^.

mTOR is a serine-threonine kinase which is related to several important aspects of mammalian cell function. The activity of mTOR is modulated through various extracellular and intracellular factors (such as amino acids, energy, hormones), in turn, mTOR changes rates of translation, transcription, protein synthesis degradation, cell signaling, metabolism, and cytoskeleton dynamics^14^. Specially, mTOR signaling pathway plays a key role in maintaining intestinal health^15,18,19^. 4EBP1, as one of the most well-known substrates of mTOR, is involved in regulation of the rate of protein synthesis^36^. Phosphorylation of 4EBP1 by mTOR promotes dissociation of 4EBP1 from Eukaryotic initiation factor 4E (EIF4E), enabling EIF4E to induce protein translation^37^. Recent studies have shown that amino acids as important signaling regulators for intestinal protein synthesis and cell growth via mTOR, could regulate downstream signal 4EBP1^15,16^. In this experiment, supplementation with Glu increased jejunal and ileal ratio of p-mTOR/t-mTOR in the LPS-challenged pigs. These results indicate that Glu activated mTOR signaling pathway, further to improve protein synthesis, which is consistent with increased intestinal RNA/DNA. We speculated that the protective effect of Glu on intestinal mTOR signaling pathway might be due to the following mechanisms. Firstly, Glu phosphorylated mTOR directly. There were reports showed that Glu activated mTOR as neurotransmitter, or the activation of both ionotropic and metabotropic glutamate receptors (mGluR) induced mTOR^14^. Glu supplementation increased the relative mRNA expression of the jejunal mucosa mGluR1 and mGluR4 in weaning piglets^35^. Secondly, Glu could be oxidized directly to produce energy which further affects mTOR. Glu has been demonstrated to be one of the major sources of energy in mammalian enterocytes via mitochondrial oxidation^21^. In addition, Glu might exert its beneficial effects through transforming into arginine and glutamine^23^. Abundant evidences have shown that arginine and glutamine participate in regulating mTOR and their downstream signals in intestine^15,38^. For example, there was study demonstrating that arginine-dependent cell survival and protein synthesis signaling in IPEC-J2 cells were mediated by mTOR^15^. Xi *et al*. reported that glutamine could regulate protein synthesis in intestinal cells through the mTOR signaling pathway^38^.

TLR4 and NOD signaling pathways are activated to defense against pathogens invading via triggering the production of pro-inflammatory cytokines and inflammatory response. In our present study, supplementation with 2.0% Glu reduced mRNA expressions of key genes in TLR4 (jejunal TLR4, MyD88, IRAK1 and TRAF6) and NOD (jejunal NOD2) signaling pathways in the pigs challenged with LPS, which is in consistent with the decreased TNF-α concentration in plasma. However, there are few studies on Glu regulating TLR4, NODs and their downstream signals. In the central nervous system, Glu is the major excitatory neurotransmitter^26^, and its metabolism is involved with inflammation^39^. So it is possible that Glu exerted a protective effect on inhibiting TLR4 and NOD signaling pathways as an excitatory neurotransmitter, further reducing inflammation in intestine. In addition, as a member of the “arginine family”, Glu can convert into other amino acids (arginine, glutamine, aspartate, asparagine, proline, ornithine and citrulline) via complex interorgan metabolism in most mammals, including the pig^23^. Glu might exert its beneficial effects through transforming into arginine, asparagine and aspartate^23^. Some previous evidence has shown that arginine, aspartate, and asparagine participate in regulating TLRs, NOD and their downstream signals in weaned piglets^40–42^.

The aberrant activation of inflammatory signaling pathways (TLR4 and NOD) elicits collateral host tissue injury^43^. In order to prevent excessive inflammatory responses, many mechanisms can negatively control inflammatory signaling pathways. So far, several negative regulators of TLR4 (such as SOCS1 and Tollip) and NOD (such as ERBB2IP and ACAP1) signaling pathways have been identified and characterized^44–46^. Our data reflected that the LPS-challenged pigs had reduced mRNA expressions of ileal ERBB2IP and Tollip, which is in agreement with the study of Wang *et al*.^40^. Our results indicate that LPS challenge down-regulated the gene expressions of intestinal negative regulators in TLR4 and NOD signaling pathways, which is in consistent with increased mRNA expressions of intestinal key genes in TLR4 and NOD signaling-related genes. Thus, the up-regulation of key genes in TLR4 and NOD signaling-related genes might be related to the decrease of their negative regulators. In the present study, supplementation with 2.0% Glu increased mRNA expression of ileal Tollip, decreased gene expression of jejunal ERBB2IP. Wu *et al*. demonstrated that Tollip down-regulated the TLR4 signaling pathway via bounding to IL-1 receptor-associated kinase (IRAK) and inhibiting IRAK phosphorylation^44^. This indicates that supplementation with Glu could inhibit the activity of IRAK through increasing the gene expression of Tollip, leading to impair the signaling from TLR4 to downstream pathways and reduce the synthesis of pro-inflammatory cytokines. Curiously, Glu inhibited mRNA expressions of ERBB2IP. It is possible that Glu inhibited TLR4 and NOD signaling pathways directly or through other non-negative regulatory pathways to reduce excessive release of inflammatory cytokines, which resulted in less mRNA expression of ERBB2IP, but this needs to be further studied.

In conclusion, supplementation with Glu alleviates intestinal damage and improves intestinal repair in LPS-challenged piglets. The protective effects of Glu on the intestine may be associated with maintaining mTOR and inhibiting TLR4 and NOD signaling pathways.

## Methods

### Animal care and experimental design

The animal experiment was carried out in accordance with the Chinese Guidelines for Animal Welfare and Experimental Protocol, and was approved by the Animal Care and Use Committee of Wuhan Polytechnic University. A total of 24 crossbred pigs (Duroc × Large White × Landrace, initial body weight of 7.02 ± 0.21 kg) were used and randomly allotted into 4 treatments. Every treatment included 6 replicate pens. Piglets were housed in 1.80 × 1.10 m^2^ pens and had free access to drinking water and their diets individually. The basal diet was formulated according to National Research Council requirements for all nutrients (Table 9)^47^. Ambient temperature was maintained at 25–27 °C.

**Table 9:** Ingredient composition of experimental diets (%, as-fed basis).

Ingredients		Nutrients level^b^	
Corn	56.40	Digestible energy (MJ/kg)	13.70
Soybean meal, 44% crude protein	22.40	Crude protein	19.20
Wheat middling	5.00	Calcium	0.89
Fish meal	3.60	Total phosphorus	0.67
Soy protein concentrate	1.40	Total aspartate + asparagine	1.65
Fat powder	2.00	Total glutamate + glutamine	2.97
Defatted milk-replacer power	3.00	Arginine	0.96
Dicalcium phosphate	1.22	Lysine	1.02
Limestone	0.94	Serine	0.90
Salt	0.34	Threonine	0.74
Alanine	1.21	Proline	1.11
Cornstarch	0.79	Glycine	0.70
Acidifier	0.20	Alanine	2.00
L-Lysine HCl, 78%	0.27	Histidine	0.51
DL-Methionine, 99%	0.10	Leucine	1.45
L-Threonine, 98%	0.08	Isoleucine	0.55
Butylated hydroquinone	0.05	Tyrosine	0.42
Vitamin and mineral premix^a^	1.00	Phenylalanine	0.71
Total	100.00	Valine	0.63

^a^Premix supplied per kg diet: retinyl acetate, 5512 IU; cholecalciferol, 2200 IU; DL-α-tocopheryl acetate, 30 IU; menadione sodium bisulfite complex, 4 mg; riboflavin, 5.22 mg; D-calcium-pantothenate, 20 mg; niacin, 26 mg; vitamin B_12_, 0.01 mg; Mn (MnSO_4_ · H_2_O), 40 mg; Fe (FeSO_4_ · H_2_O), 75 mg; Zn (ZnSO_4_ · 7H_2_O), 75 mg; Cu (CuSO_4_ · 5H_2_O), 100 mg; I (CaI_2_), 0.3 mg; Se (Na_2_SeO_3_), 0.3 mg.

^b^The nutrients level was analyzed value except digestible energy which is calculated value.

The 4 treatments were set as follows: (1) control (basal diet + 0.9% NaCl solution); (2) LPS (basal diet + *E*. *coli* LPS (*E*. *coli* serotype 055: B5; Sigma Chemical Inc., St Louis, MO, USA); (3) LPS + 1.0% Glu (1.0% Glu diet + LPS); (4) LPS + 2.0% Glu (2.0% Glu diet + LPS).

The doses of Glu (purity > 99%; Amino Acid Bio-Chemical, Wuhan, China) were used according to Rezaei’s studies^24^. In order to obtain isonitrogenous diets, 1.21, 0.61 and 0% alanine (purity > 99%; Amino Acid Bio-Chemical, Wuhan, China) were added to the control, 1.0% Glu and 2.0% Glu diets, respectively. On day 28, pigs were injected intraperitoneally with either LPS at 100 μg/kg body weight or the same amount of 0.9% (w/v) saline. The LPS was dissolved in sterile 0.9% NaCl solution (500 mg LPS /L saline).

### Sample collection

At 4 h post-injection, we collected blood samples into heparinized vacuum tubes from the anterior vena cava and centrifuged (3000 r/min, 15 min, 4 °C) to separate plasma. Plasma was stored at −80 °C until analysis of TNF-α. Following blood collection, piglets were harvested using pentobarbital at 80 mg/kg body weight, and 2 gut samples were taken from the mid-jejunum (3 cm and 10 cm) as well as mid-ileum (3 cm and 10 cm). The collection methods of intestinal samples were referred to Liu *et al*.^4^. The 3 cm sections were rinsed gently with 0.9% ice-cold saline, and then kept in 4% paraformaldehyde in PBS waiting to determine intestinal morphology. The 10 cm sections were opened and luminal contents were removed and rinsed. Mucosal samples were collected using a sterile glass slide, then immediately packaged and put in liquid nitrogen to froze, and stored at −80 °C for subsequent analysis.

### Intestinal morphology

Intestinal samples were dehydrated, embedded in paraffin, sectioned, and stained with haematoxylin and eosin after fixation in 4% paraformaldehyde for 24 h^4^. Then jejunal and ileal samples were analyzed on histologic slides by a microscope (Olympus, Japan) at 10× magnification using Image-Pro Plus software. Villus height and crypt depth were measured referred to the previous methods reported by Nunez *et al*.^48^. Briefly, a minimum of 10 well-oriented and intact villi were chosen. Villus height was defined as the distance from the villus tip to crypt mouth, and crypt depth was measured from crypt mouth to base.

### Concentrations of mucosal protein, DNA and RNA

Intestinal mucosal samples were homogenized, and then centrifuged to harvest the supernatant for determining the concentrations of protein, DNA and RNA. The analysis procedures were performed according to the previous reports^4^. Protein concentration of mucosal homogenates was determined by a detergent-compatible protein assay (Bio-Rad Laboratories, Hercules, CA, USA) and using bovine serum albumin as standards. Mucosal DNA content was measured by a fluorometric assay. Mucosal RNA content was measured by spectrophotometry with a modified Schmidt-Tannhauser method.

### Disaccharidase activities

Disaccharidase activities in the supernatant of intestinal mucosa were determined referred to previous study reported by Hou *et al*.^49^ using glucose kits (#A082–1 for lactase, #A082-2 for sucrase and #A082-3 for maltase; Nanjing Jiancheng Bioengineering Institute, Nanjing, China).

### Plasma TNF-α concentration

The TNF-α concentration in plasma was measured by using a commercially available porcine ELISA kit (R&D Systems, Minneapolis, MN, USA, REF: PTA00)^40^.

### mRNA abundance analysis

Intestinal total RNA was extracted using RNAiso Plus (TaKaRa, 9108/9109, Dalian, China), and dissolved in RNase-free water. A total of 1 μg of RNA, as templates, was reverse-transcribed into cDNA using the PrimeScript™ RT reagent kit with gDNA Eraser (TaKaRa, RR047A, Dalian, China). The reaction was carried out for 15 min at 37 °C, 5 sec at 85 °C, ∞ at 4 °C. RT-PCR was performed on an ABI 7500 Real Time PCR system (Applied Biosystems, Foster City, CA, USA) using SYBR® Premix Ex Taq™ (TaKaRa, Dalian, China). We used specific primers to measure the mRNA abundance. GAPDH was used as a housekeeping gene to normalize the target gene transcript levels. Primer sequences are listed in Table 10. The relative mRNA expression of genes was calculated as a ratio of the target gene to the control gene depending on the the 2^−ΔΔCT^ method^4^.

**Table 10:** Primer sequences used for real-time PCR.

Gene	Forward (5′-3′)	Reverse (5′-3′)	Product length (bp)	Accession numbers
TLR4	TCAGTTCTCACCTTCCTCCTG	GTTCATTCCTCACCCAGTCTTC	166	GQ503242.1
MD2	TGCAATTCCTCTGATGCAAG	CCACCATATTCTCGGCAAAT	227	NM_001104956.1
CD14	CGTTTGTGGAGCCTGGAAG	TGCGGATGCGTGAAGTTG	226	NM_001097445.2
LBP	GAACACAGCCGAATGGTCTAC	GGAAGGAGTTGGTGGTCAGT	151	NM 001128435.1
MyD88	GATGGTAGCGGTTGTCTCTGAT	GATGCTGGGGAACTCTTTCTTC	148	AB292176.1
IRAK1	CAAGGCAGGTCAGGTTTCGT	TTCGTGGGGCGTGTAGTGT	115	XM_003135490.1
TRAF6	CAAGAGAATACCCAGTCGCACA	ATCCGAGACAAAGGGGAAGAA	122	NM_001105286.1
RP105	CGAGGCTTCTGACTGTTGTG	GGTGCTGATTGCTGGTGTC	245	AB190767.1
SOCS1	GCGTGTAGGATGGTAGCA	GAGGAGGAGGAGGAGGAAT	101	NM_001204768.1
Tollip	GCAGCAGCAACAGCAGAT	GGTCACGCCGTAGTTCTTC	133	AB490123.1
SIGIRR	ACCTTCACCTGCTCCATCCA	TTCCGTCATTCATCTCCACCTC	205	AB490122.1
NOD1	CTGTCGTCAACACCGATCCA	CCAGTTGGTGACGCAGCTT	57	AB187219.1
NOD2	GAGCGCATCCTCTTAACTTTCG	ACGCTCGTGATCCGTGAAC	66	AB195466.1
RIPK2	CAGTGTCCAGTAAATCGCAGTTG	CAGGCTTCCGTCATCTGGTT	206	XM_003355027.1
NF-κB	AGTACCCTGAGGCTATAACTCGC	TCCGCAATGGAGGAGAAGTC	133	EU399817.1
ERBB2IP	ACAATTCAGCGACAGAGTAGTG	TGACATCATTGGAGGAGTTCTTC	147	GU990777.1
ACAP1	GAAGCCGAAGTGTCCGAATT	AGGTCACAGATGCCAAGAATG	125	XM_003358258.2
GAPDH	CGTCCCTGAGACACGATGGT	GCCTTGACTGTGCCGTGGAAT	194	AF017079.1

### Protein abundance analysis

The protein abundance of intestinal mucosa was determined according to the procedures of Wang *et al*.^34^. Specific primary antibodies included rabbit anti-claudin-1 (1:1000) from Invitrogen (Invitrogen Technology Inc., Danvers, MA, USA), rabbit anti-mTOR (1:1000), rabbit anti-phosphorylated mTOR (Ser^2448^; 1:1000), rabbit anti-4EBP1 (1:1000), rabbit anti-phosphorylated 4EBP1 (Thr^70^; 1:1000) from Cell Signaling (Cell Signaling Technology Inc., Danvers, MA, USA), and mouse anti-β-actin (1:10,000) from Sigma Aldrich (Sigma Aldrich Inc., St. Louis, MO, USA). The protein expression of claudin-1 was expressed as the ratio of claudin-1/β-actin. Phosphorylated forms of mTOR and 4EBP1 were normalized against the total protein content of each protein.

### Statistical analysis

Data were analyzed by the GLM procedure of SAS (SAS Inst. Inc., Cary, NC, USA). The differences among group means were compared using Duncan Multiple Comparison based on the variance derived from ANOVA. Individual piglet was used as an experimental unit for the data. Results were expressed as means and pooled SEM. Significant differences were considered at p < 0.05.
